# Combination of the oral histone deacetylase inhibitor resminostat with oncolytic measles vaccine virus as a new option for epi-virotherapeutic treatment of hepatocellular carcinoma

**DOI:** 10.1038/mto.2015.19

**Published:** 2015-10-07

**Authors:** Benjamin Ruf, Susanne Berchtold, Sascha Venturelli, Markus Burkard, Irina Smirnow, Tanja Prenzel, Stefan W Henning, Ulrich M Lauer

**Affiliations:** 1 Department of Internal Medicine I, University Hospital Tuebingen, Tuebingen, Germany; 2 4SC AG, Planegg-Martinsried, Germany

## Abstract

Epigenetic therapies such as histone deacetylase inhibitors (HDACi) not only have the capability to decrease tumor cell proliferation and to induce tumor cell death but also to silence antiviral response genes. Here, we investigated whether the combination of an oncolytic measles vaccine virus (MeV) with the novel oral HDACi resminostat (Res), being in clinical testing in patients with hepatocellular carcinoma (HCC), results in an enhanced efficacy of this epi-virotherapeutic approach compared to any of the two corresponding monotherapies. When testing a panel of human hepatoma cell lines, we found (i) a significantly improved rate of primary infections when using oncolytic MeV under concurrent treatment with resminostat, (ii) a boosted cytotoxic effect of the epi-virotherapeutic combination (Res + MeV) with enhanced induction of apoptosis, and, quite importantly, (iii) an absence of any resminostat-induced impairment of MeV replication and spread. Beyond that, we could also show that (iv) resminostat, after hepatoma cell stimulation with exogenous human interferon (IFN)-β, is able to prevent the induction of IFN-stimulated genes, such as IFIT-1. This finding outlines the possible impact of resminostat on cellular innate immunity, being instrumental in overcoming resistances to MeV-mediated viral oncolysis. Thus, our results support the onset of epi-virotherapeutic clinical trials in patients exhibiting advanced stages of HCC.

## Introduction

In response to viral pathogens, mammalian cells have developed an arsenal of innate immunity factors to prevent viral infections, with a central role assigned to the interferon (IFN) system.^1^ Virus-derived pathogen-associated molecular patterns are detected by, *e.g.*, cytoplasmic viral nucleic acid sensors such as RIG-I and MDA5 (ref. 2) or membrane-associated Toll-like receptors,^3^ with subsequent activation of downstream NF-κB signaling^4^ or IRF-3/IRF-7 binding to the IFN promoter site,^5^ resulting in transcription and secretion of type I IFNs. Autocrine- and paracrine-produced IFN binds to the membrane-associated IFN receptor with consecutive activation of the downstream JAK/STAT signaling pathway.^6^ As a result, transcription of IFN-stimulated genes (ISG) is induced, such as IFN-induced proteins with tetratricopeptide repeats (IFIT family), establishing an antiviral state within the infected cell as well as in noninfected bystanding cells.^7^ Recently, it was shown that measles virus (MeV) vaccine strains such as the Edmonston strain of MeV, but not wild-type MeV, induce production of IFN-β, *e.g.*, via IRF-3 activation.^8,9^ Since MeV-based virotherapeutics are generated on backbones of MeV vaccine strains,^10,11^ MeV-induced production of IFN-β could have strong implications on rates of primary infection, replication, and spread of MeV in tumor tissues, thereby constituting a severe limitation to MeV-based oncolytic virotherapy approaches. These limitations might be overcome by combining oncolytic viruses^12^ with epigenetic compounds such as histone deacetylase inhibitors (HDACi) which are able to “attenuate” the antiviral defense mechanisms in a transient manner.^13^ Recently, resensitization to vesicular stomatitis virus (VSV)–induced oncolysis by HDACi like entinostat (MS-275) and vorinostat (SAHA) has been demonstrated to result in a significant improvement of viral replication.^14^ Interestingly, entinostat combined with a virotherapeutic prime-boost using vectors of VSV and adenovirus origin, both expressing human dopachrome tautomerase, were found to suppress primary immune responses but to enhance secondary immune responses, resulting in a prolonged survival in a murine melanoma model.^15^ In another experimental system, vaccinia virus replication and spread were found to be boosted by combination with the HDACi trichostatin A (TSA).^16,17^ A similar pattern was found for the combination of herpes simplex virus (HSV) and the HDACi valproic acid (VPA)^18^ revealing that VPA treatment impaired recruitment of immune cells as well as innate immunity signaling.^19^ Replication of HSV was found to be intensified when employing a whole panel of different HDACi,^20^ and additional antiangiogenic effects were identified for the combination of TSA plus HSV.^21^ Another virotherapeutic vector, parvovirus H-1PV, led to additional functional insights on potential combinational mechanisms: addition of VPA was found to increase acetylation and thereby cytotoxicity of the NS1 protein of H-1PV.^22^ HDAC inhibition in combination with adenovirus results in the upregulation of CAR, a membrane receptor for coxsackie and adenovirus subgroups.^23^ However, opposite outcomes such as VPA-mediated inhibition of both adenovirus replication and spread have also been reported,^24^ indicating that every individual combination of HDACi and oncolytic virus has to be investigated in detail.

Resminostat constitutes a novel oral HDACi with selectivity for class I and IIb HDAC isoenzymes and has undergone clinical evaluation in a phase 1/2 clinical trial in patients with advanced stage HCC (NCT00943449). *In vitro* resminostat was shown to induce apoptosis in concentrations above 2.5 μmol/l, whereas lower concentrations resulted in a proliferation stop and cell cycle arrest.^25^ This profile proposes resminostat as an interesting partner for novel epi-virotherapeutic concepts in the combinatorial treatment of patients exhibiting advanced stages of HCC. Accordingly, we here investigated whether the combination of an oncolytic measles vaccine virus with resminostat results in an enhanced efficacy of this epi-virotherapeutic approach when compared to any of the two corresponding monotherapies.

## Results

### Antitumoral activities of resminostat and MeV on human hepatoma cell lines

Combinations of various epigenetic compounds with oncolytic viruses have been shown to result in the enhancement of therapeutic efficacy, encouraging further investigation of novel combinatorial epi-virotherapeutic settings. In this context, we have tested the antitumoral potency of either resminostat, a novel oral HDACi,^25^ or MeV-super-cytosine deaminase (SCD), a prototypic suicide gene-armed measles vaccine virotherapeutic,^11^ in a commonly used panel of human hepatoma cell lines (HepG2, Hep3B, PLC/PRF/5).

**Figure 1:**
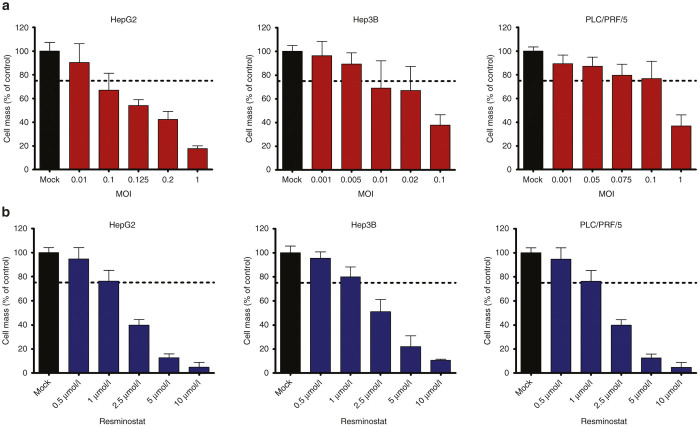
Remaining tumor cell masses after single (monotherapeutic) treatment with either MeV-SCD or resminostat. (**a**) Human hepatoma cell lines HepG2, Hep3B, and PLC/PRF/5 were infected with the prototypic suicide gene-armed measles vaccine-based virotherapeutic MeV-SCD at the indicated multiplicities of infection (MOIs). Ninety-six hours postinfection (hpi), the remaining hepatoma cell masses were determined by a sulforhodamine B (SRB) viability assay. (**b**) Human hepatoma cell lines HepG2, Hep3B, and PLC/PRF/5 were treated with increasing concentrations of resminostat. At 96 hours, remaining hepatoma cell masses were determined by an SRB assay. Displayed are means and SDs of three independent experiments each carried out in quadruplicates. Dotted lines indicate the 75% threshold of remnant tumor cell masses at 96 hours posttherapeutic intervention. MOCK, untreated control; Res, resminostat.

For this purpose, human hepatoma cells were infected in a first step with different multiplicities of infection (MOIs), ranging for HepG2 cells from MOI 0.01 to 1, for Hep3B cells from MOI 0.001 to 0.1, and for PLC/PRF/5 cells from MOI 0.001 to 1 (Figure 1a). Then, at 96 hours postinfection (hpi), remaining hepatoma cell masses were quantified by a sulforhodamine B (SRB) viability assay. As a result, susceptibilities of these hepatoma cell lines to MeV-SCD–mediated oncolysis were found to vary within a large range (Figure 1a). Thus, in subsequent experiments, we used different (adjusted) MOIs for hepatoma cell lines HepG2 (MOI 0.1), Hep3B (MOI 0.01), and PLC/PRF/5 (MOI 0.075). On this basis, remnant tumor cell masses of ≈75% (Figure 1a, dotted lines) were ensured for monotherapy with MeV-SCD. This ≈75% threshold was highly instrumental in providing still sufficient amounts of viable hepatoma cells to be killed in later testing scenarios, in which we applied the epi-virotherapeutic combination of resminostat plus MeV-SCD (Res + MeV).

In a second step, we also investigated the monotherapeutic cytotoxic potential of resminostat on human hepatoma cell lines. For this purpose, HepG2, Hep3B, and PLC/PRF/5 cells were incubated for 96 hours with increasing concentrations of resminostat (ranging from 0.5 to 10 µmol/l; Figure 1b). As a result, resminostat was found to reduce hepatoma cell masses being residual at 96 hours in a dose-dependent manner (Figure 1b). Again, we set out to attain a residual hepatoma cell mass of ≈75% also in the monotherapeutic use of resminostat (Figure 1b, dotted lines), which could be easily achieved by applying a uniform resminostat concentration of 1 µmol/l for all three hepatoma cell lines used.

### Boosted cytotoxic/oncolytic effect of the epi-virotherapeutic combination treatment

**Figure 2:**
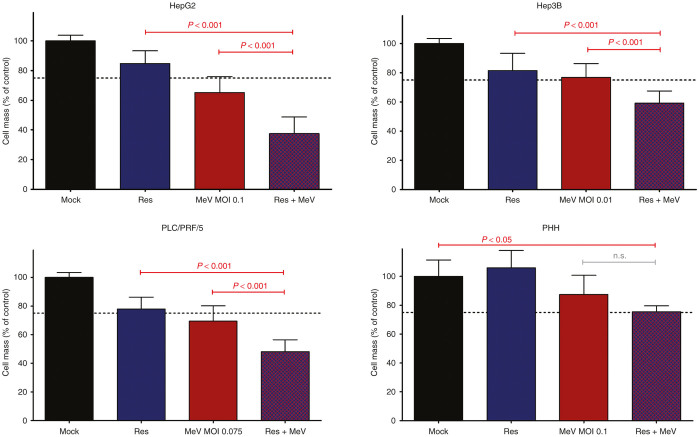
Boosted cytotoxic effects obtained by epi-virotherapeutic co-treatment with resminostat and MeV-SCD. Human hepatoma cell lines HepG2, Hep3B, and PLC/PRF/5 and nonmalignant primary human hepatocytes (PHHs; lower right panel) were infected with MeV-SCD (at adjusted MOIs) and cotreated with resminostat (1 μmol/l) at 3 hpi. Endpoint measurements were performed at 96 hpi using the SRB viability assay. Displayed are means and SDs of at least three independent experiments for hepatoma cells and one experiment for PHH cells, each carried out in quadruplicates; *P* values of one-way ANOVA with a Tukey posttest. hpi, hours postinfection; MeV, suicide gene-armed measles vaccine-based virotherapeutic MeV-SCD; MOI, multiplicity of infection; n.s., not significant; Res, resminostat.

In a next step, we investigated the specific combinatorial epi-virotherapeutic potential of HDAC inhibition plus virus-mediated oncolysis (Res + MeV). For this purpose, hepatoma cells HepG2, Hep3B, and PLC/PRF/5 were first infected with MeV-SCD (using threshold-adjusted MOIs as described above). At 3 hpi, resminostat was added also in a threshold-adjusted manner (1 µmol/l). As a result, boosted combined cytotoxic/oncolytic effects were observed in all three human hepatoma cell lines (Figure 2) when compared with any of the two corresponding single agent/monotherapeutic treatment regimens, leading to a significant reduction of tumor cell masses as being quantified by SRB assays (purple bars (combi) versus blue/red bars (*mono*)). In detail, for HepG2 cells, we found a reduction of hepatoma cell masses for the combinatorial setting down to 37.6% compared to 84.7% (resminostat alone) and 65.2% (MeV-SCD single-agent treatment). These results were confirmed exemplarily for HepG2 tumor cells. For this purpose, tumor cell viabilities were exemplarily determined also by the CellTiter-Blue assay which quantifies membrane integrities/viable tumor cells via their capacity to metabolize a specific substrate, *i.e.*, resazurin. As a result, HepG2 tumor cell viability was found to decrease in the combinational setting (Res + MeV) to 49.2% compared to monotherapies exhibiting reductions of only 62.5% for MeV-SCD and only 80.4% for resminostat, respectively (see Supplementary Figure S1A). Furthermore, real-time monitoring of proliferation with the xCELLigence system was used to gain additional insight about the kinetics of tumor cell (HepG2) growth inhibition for the combinational setting (Res + MeV) compared to the monoagent treatment (Res or MeV) over a period of 120 hours after treatment. As a result, combination of MeV-SCD and resminostat induced the strongest diminution of impedance of the cell layer (displayed as normalized cell indices in Supplementary Figure S1C) in comparison to the respective MeV or resminostat monotherapy. Finally, Supplementary Figure S1D displays normalized cell indices (generated by the xCELLigence system) for the above-mentioned treatment regimens 120 hours after treating the HepG2 tumor cells. Here, a trend favoring the Res + MeV approach was found (although requirements of statistic testing were not met). Taken together, these additional data confirmed the superior effect of the MeV + Res combination treatment versus any of the monotherapeutic approaches.

In Hep3B cells, hepatoma cell masses were reduced to 59.1% (combi) whereas reduction to only 81.4% (resminostat) or 76.8% (MeV-SCD) could be achieved in monotherapeutic approaches. PLC/PRF/5 hepatoma cells reached a 48.1% residual tumor cell mass (combi) compared to 77.8% (resminostat) and 69.4% (MeV-SCD) in monotherapeutic approaches. Interestingly, these findings could not be confirmed in (nonmalignant) primary human hepatocytes (PHH), where addition of resminostat to MeV-SCD–infected primary cells did not further reduce PHH cell masses. Although addition of Resminostat to MeV-infected cells did not reduce PHH cell masses (Figure 2, lower right panel, lane 2), combination treatment (MeV + Res; (Figure 2, lower right panel, lane 4) did significantly affect PHH viability compared to the untreated control (Figure 2, lower right panel, lane 1). This effect seems to be largely dependent on MeV infection as Resminostat treatment alone had been found to have no impact on PHH viability (Figure 2, lower right panel, lane 3).

Thus, a proof-of-principle has been provided for the profound antitumoral effects of a novel combination therapy based on the oral HDACi resminostat combined with oncolytic measles vaccine virus MeV-SCD. Since this specific epi-virotherapeutic combination (Res + MeV) potentially could define a new therapeutic option for HCC patients, we further investigated molecular mechanisms possibly underlying the observed boosted antitumoral effect in detail.

### Resminostat-mediated enhancement of primary infection rates in hepatoma cell lines

**Figure 3:**
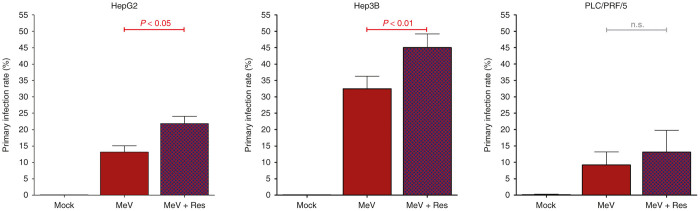
Resminostat is able to enhance rates of primary infection of human hepatoma cells by measles vaccine virotherapeutics. Human hepatoma cell lines HepG2, Hep3B, and PLC/PRF/5 were infected with MeV-GFP at a standardized MOI of 1 and cotreated with resminostat (1 μmol/l) from 3 hpi on. At 24 hpi, quantitative differences in primary infection rates (defined as the percentage of infected cells (%)) were determined by flow cytometry. Displayed are means and SDs of three independent experiments. *P* values of one-way ANOVA with a Tukey posttest. ANOVA, analysis of variance; hpi, hours postinfection; MeV, virotherapeutic vector MeV-GFP encoding the green fluorescent protein marker gene; MOCK, untreated control; MOI, multiplicity of infection; n.s., not significant; Res, resminostat.

To further investigate the underlying molecular mechanisms of the epi-virotherapeutic boosted antihepatoma effect, primary infection rates were determined using flow cytometry. For this purpose, HepG2, Hep3B, and PLC/PRF/5 cells were first infected with a derivative measles vaccine virus encoding the green-fluorescent marker protein (MeV-GFP) at a nonadjusted MOI of 1, and then cotreated with resminostat (1 µmol/l). As a result, the percentage of infected hepatoma cells being determined at 24 hpi (a time point at which replication of MeV has not yet resulted in release of progeny virus particles and secondary infections of target cells) was found to be enhanced in all three human hepatoma cell lines in the combination groups (Res + MeV; Figure 3, purple bars) compared to the solely MeV-GFP–infected groups (Figure 3, red bars). In detail, addition of resminostat elevated the percentage of MeV-GFP-infected HepG2 cells at 24 hpi from 13.2 to 21.9%, in Hep3B cells from 32.5 to 45.0%, both in a statistically significant manner; only in PLC/PRF/5 cells addition of resminostat was found to result in a minor rise of MeV-GFP–infected cells from 9.2 to 11.1%, which was statistically insignificant. Interestingly, we found that all primary infection rates correlated quite well with the susceptibility of the hepatoma cell lines to MeV-mediated oncolysis (Figure 1a). Notably Hep3B cells, exhibiting the most distinct MeV-mediated oncolytic effect already at quite low MOIs (*i.e.*, MOI 0.01), showed the highest primary infection rate, indicating that the efficiency of hepatoma cell infection within the first 24 hours could be crucial to oncolytic tumor cell destruction. In this context, it is of interest that resminostat was found to be able to induce an enhancement of primary infection, at least partially contributing to a boosted oncolytic effect being determined by the endpoint measurement of tumor cell viabilities (*i.e.*, at 96 hpi).

### Addition of resminostat does not impair MeV replication and spread

**Figure 4:**
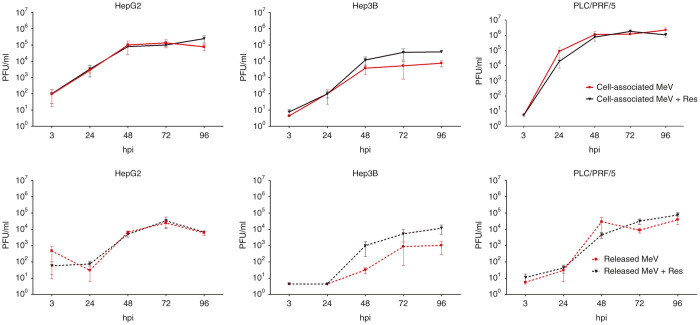
Resminostat does not impair replication of MeV-SCD and subsequent spread of progeny viral particles. At 3, 24, 48, 72, and 96 hpi, cell lysates (comprising cell-associated MeV particles; upper panels) and supernatants (comprising MeV particles being released into cell culture medium; lower panels) were sampled either from solely MeV-SCD infected human hepatoma cells (HepG2, Hep3B, PLC/PRF/5; employing adjusted MOIs) or obtained after epi-virotherapeutic cotreatment with resminostat applied at a concentration of 1 µmol/l (MeV + Res). Vero cells were used for virus titrations, and results were converted into PFU/ml. Results of solely MeV-infected cells (red lines) are displayed along with results of MeV + Res cotreated cells (purple lines). Solid lines are representative for the quantification of cell-associated viral particles (upper row), whereas dotted lines are used to highlight viral particles being released into hepatoma cell supernatants (lower row). Means and SEM are shown for three independent experiments. hpi, hours postinfection; PFU, plaque forming unit; Res, resminostat.

To gain further insight into the kinetics of MeV-SCD replication and spread under the influence of resminostat, we worked out viral growth curves in absence/presence of resminostat (Figure 4). For this purpose, hepatoma cells were infected with MeV-SCD at adjusted MOIs as being defined before (HepG2: 0.1; Hep3B: 0.01; PLC/PRF/5: 0.075). At 3 hpi, resminostat (1 µmol/l) was added, and MeV replication was quantified at 3, 24, 48, 72, and 96 hpi. As a result, we did not find any impairment of MeV-SCD replication and spread in HepG2 and PLC/PRF/5 hepatoma cell cultures (Figure 4, panels to the left and right). Interestingly, in Hep3B cells (Figure 4, panels in the middle), we found an enhancement of MeV progeny virus production by factor 10 in the presence of resminostat (both for cell associated as well as for MeV particles released into cell culture supernatants), but these findings turned out to not be statistically significant. Therefore, boosted antitumoral effects of the epi-virotherapeutic combination treatment (Res + MeV) seem to be largely independent of a putative resminostat-mediated enhancement of viral replication and spread.

### Resminostat-induced downregulation of zfp64 in MeV-infected hepatoma cells

**Figure 5:**
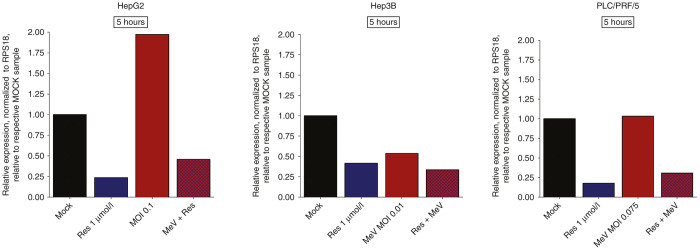
Expression levels of zfp64 (pharmacodynamic biomarker for resminostat activity) in human hepatoma cell lines after epi-virotherapeutic (Res + MeV) treatment: HepG2, Hep3B, and PLC/PRF/5 cells were infected with MeV-SCD at indicated MOIs and cotreated with resminostat (1 µmol/l) from 3 hpi on. RNA was isolated after 5 hours of incubation with resminostat. Then, zfp64 expression levels were determined using RT-qPCR. Values were normalized to the housekeeping gene RPS18 (ribosomal-protein S18), and relative expression is displayed compared to corresponding control samples (MOCK). Data of one experiment are shown. Res, resminostat.

To provide proof for an unimpaired activity of resminostat in the course of MeV-based infections of human hepatoma cells, we determined expression levels of zinc finger protein 64 (zfp64), which functions as a well-established surrogate parameter for the pharmacological activity of resminostat, in absence/presence of MeV-SCD. As shown before,^26^ when resminostat (1 µmol/l) was applied alone (*i.e.*, in absence of MeV-SCD), it was found to inhibit zfp64 mRNA production quite effectively in all three human hepatoma cell lines at early time points, *i.e.*, at 5 hours after addition of resminostat (Figure 5, blue bars). Interestingly, when resminostat (1 µmol/l) was added subsequent to infections with MeV-SCD (employing adjusted MOIs), again lower zfp64 mRNA expression levels were observed in all three hepatoma cell lines when being compared to untreated controls (Figure 5, purple bars). In contrast, monotherapeutic applications of MeV-SCD (Figure 5, red bars) were found (i) to enhance zfp64 expression levels in HepG2 cells, (ii) to reduce zfp64 expression levels in Hep3B cells, and (iii) to not change zfp64 expression levels in PLC/PRF/5 cells (when compared to untreated controls (MOCK), respectively). Thus, resminostat-induced downregulation of zfp64 expression was found to take place also in the course of epi-virotherapeutic cotreatment (Res + MeV) of human hepatoma cells, indicating an unimpaired effect of resminostat in this specific epi-virotherapeutic context, a finding which is highly essential for the further clinical development of this combinatorial approach.

### Epi-virotherapeutic treatment (Res + MeV) enlarges apoptosis of hepatoma cells

**Figure 6:**
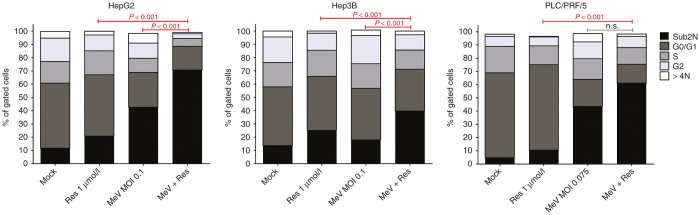
Cell cycle profiles of human hepatoma cells undergoing epi-virotherapeutic cotreatment with resminostat and MeV-SCD: human hepatoma cell lines HepG2, Hep3B, and PLC/PRF/5 were infected with MeV-SCD (MeV) or mock infected (MOCK) and resminostat (Res) was added at 3 hpi or not. Intracellular DNA was stained with propidium iodide (PI) at 96 hpi and measured by flow cytometry. Combinatorial treatment (Res + MeV) was found to enhance the population of hypoploid/apoptotic cells (black vertical bars: sub2N) compared to corresponding single agent treatments (Res or MeV); concurrently, fractions of proliferating cells (represented in cell cycle phases S and G_2_) were found to diminish. Means of three independent experiments carried out in duplicates or triplicates are shown. *P* values of one-way ANOVA with a Tukey posttest.

To gain additional insight into boosted antitumoral effects of this specific epi-virotherapeutic (Res + MeV) treatment, we also analyzed cell cycle profiles of our human hepatoma cell panel. Again, HepG2, Hep3B, and PLC/PRF/5 cells were infected with MeV-SCD or mock infected and resminostat (1 µmol/l) was added at 3 hpi or not. At 96 hpi, intracellular DNA was stained with propidium iodide, and the percentage of hepatoma cells within each phase of the cell cycle was determined via flow cytometry (Figure 6). Single-agent treatment with resminostat led to a slight increase of the sub2N fraction of hepatoma cells with hypoploid DNA content, indicating intracellular DNA fragmentation as a consequence of an ongoing apoptotic program (HepG2 (control/Res): 11.6%/20.8%; Hep3B: 13.6%/25.2%; PLC/PRF/5: 6.1%/11.9%). In contrast, infections with MeV-SCD (MeV) again being performed at adjusted MOIs (HepG2: 0.1; Hep3B: 0.01; PLC/PRF/5: 0.075) were found to augment the sub2N fraction (HepG2: 42.6%; Hep3B: 17.9%; PLC/PRF/5: 45.0%). Most interestingly, combinational epi-virotherapeutic treatment (Res + MeV) was found to further increase the rates of apoptotic cells (HepG2: 70.6%, *P* < 0.001; Hep3B: 39.7%, *P* < 0.001; PLC/PRF/5: 62.7%, *P* > 0.05). As a second (confirmatory) approach for the evaluation of apoptosis (performed exemplarily in HepG2 cells), we employed the tetramethylrhodamine ethyl ester (TMRE) assay which determines the breakdown of mitochondrial transmembrane potential in tumor cells (see Supplementary Figure S1B). As a result, the percentage of TMRE-negative (and therefore apoptosis-positive) cells at baseline (mock) was as high as 34.5% and showed a mean of 30.8% after treatment with resminostat. In contrast, infection with MeV-SCD increased the rate of tumor cells exhibiting a loss of mitochrondiral transmembrane potential to a mean of 75.3%; further addition of resminostat (Res + MeV) led to a rate of 89.0% TMRE-negative cells (not sufficing statistical testing, but demonstrating a trend toward an even enhanced rate of tumor cell apoptosis). Overall, the epi-virotherapeutic combination therapy showed a substantial induction of apoptosis in all human hepatoma cell lines investigated, leaving only few tumor cells capable to proliferate.

### Resminostat impedes IFIT-1 expression after exogenous IFN-β stimulation

**Figure 7:**
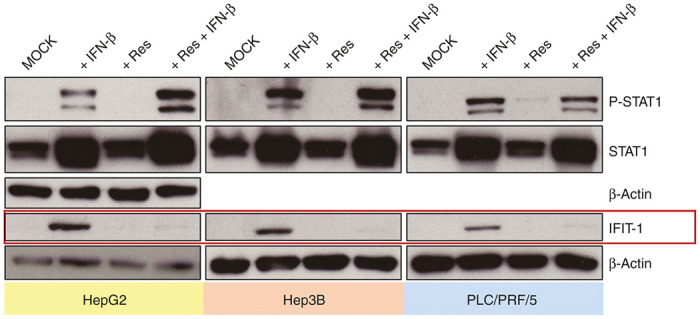
IFN-β–induced expression of IFIT-1 is suppressed by resminostat, whereas phosphorylation of STAT1 is not. HepG2, Hep3B, and PLC/PRF/5 cells were first stimulated with human IFN-β for 24 hours or left unstimulated and then treated with resminostat (5 µmol/l) or left untreated. IFIT-1 expression as well as phosphorylation and expression of STAT1 were analyzed by immunoblotting. β-Actin was used as a loading control. Shown are representative blots of three independent experiments. Res, resminostat.

To investigate potentially immunomodulating effects of resminostat on the IFN pathway, being important for the innate immune defense against infections with virotherapeutics, modulations of IFIT-1 expression and STAT1 phosphorylation were analyzed by immunoblotting. Notably, human hepatoma cell lines HepG2, Hep3B, and PLC/PRF/5 were found to be unable to produce detectable amounts of endogenous IFN-β neither at baseline (MOCK) nor after infection with MeV-SCD when using adjusted (low) MOIs (data not shown). Therefore, we had to prestimulate hepatoma cells with exogenous human IFN-β (1,000 U/ml) for 24 hours, which then led to a significant induction of expression of both IFIT-1 and STAT1 and its phosphorylation (P-STAT1) in all three hepatoma cell lines (Figure 7; lane 2 in all panels). In unstimulated/untreated controls, we found no baseline expression of IFIT-1, and no detectable amounts of P-STAT1 (Figure 7; lane 1 in all panels). As expected, treatment with resminostat (5 μmol/l) alone neither induced IFIT-1 expression nor phosphorylation of STAT1 (Figure 7; lane 3 in all panels). However, when adding resminostat (5 μmol/l) on IFN-β prestimulated (1,000 U/ml) hepatoma cells, a profound suppression of IFIT-1 expression was observed in HepG2, Hep3B, and PLC/PRF/5 hepatoma cells, but no alteration in the phosphorylation status of STAT1 (Figure 7; lane 4 in all panels).

## Discussion

Despite recent improvements in the treatment of advanced stage HCC, clinical outcome for patients in late stages of this cancer is still poor, and therefore, further improvements of therapy modalities are urgently required.

Here, we investigated the potential benefit of a new epi-virotherapeutic approach, combining a novel HDACi (resminostat^25^) with a state-of-the-art oncolytic measles vaccine virus (MeV-SCD^27^) in a panel of three human hepatoma cell lines. We found all hepatoma cell lines to be primarily susceptible to infection with MeV-SCD (defined by a remnant tumor cell mass of <50% at 96 hpi at MOI 1 (ref. 27)), but susceptibility to virus infection was found to vary by a factor of 10 between the different hepatoma cell lines. Treatment with resminostat alone (monotherapeutic approach) resulted in a coincided dose-dependent reduction of tumor cell masses in all three hepatoma cell lines. However, coadministration of resminostat and MeV-SCD (Res + MeV) resulted in a potentiated oncolysis/cytotoxic effect warranting further investigations on this combinational therapy regimen. In contrast, in nonmalignant PHHs, no enhancement of cell mass reduction was found when comparing the epi-virotherapeutic approach (Res + MeV) to any of the two monotherapeutic modalities, suggesting the combinational approach to be safe for (nonmalignant) hepatocytes and possibly also for other nontransformed cells. However, when comparing mock treatment of PHH cells with the combinational (Res + MeV) treatment setting of PHH cells, a reduced PHH viability was found (Figure 2, lower right panel). Of note, such MeV-SCD–triggered effects on *in vitro* cultured nonmalignant PHHs have already been found before (our unpublished data). Of note and in contrast to these *in vitro* findings, *in vivo* experiments in nude mice,^28,29^ in transgenic mice, as well as in macaques^30^ were overall well tolerated without revealing any safety concerns (*e.g.*, no rise in liver enzymes). Beyond that, results of published clinical trials using measles vaccine viruses (*e.g.*, refs. 31–33) exhibited excellent safety profiles not indicating induction of any organ problems such as liver failure.

We previously have demonstrated that resistance phenomena to MeV-SCD–based oncolysis could be overcome by increasing the MOI of MeV-SCD or by employing the suicide gene function of MeV-encoded SCD, which results in converting the nontoxic antifungal prodrug 5-FC to the well-known cytotoxic drug 5-FU.^11,27,28,34^ Nevertheless, increasing the dosage of administered viral vectors in patients is yet limited due to constraints in the respective manufacturing processes and as those oncocytotoxic agents face various biological barriers consisting, *e.g.*, of the host immune system as well as the tumor microenvironment, it is essential to enhance oncolytic virotherapy (OV) potency by prudent combination strategies.

In our epi-virotherapeutic combination setting, enhanced hepatoma cell mass reduction was associated with a boosted rate of cells with hypoploid intracellular DNA content indicating an ongoing apoptotic program. Previous work by others has confirmed this mechanism of action when combining HDACi with OVs. The combination of, *e.g.*, either entinostat (MS-275) or vorinostat (SAHA) together with a VSV-based virotherapeutic vector was found to enhance intrinsic apoptotic pathways, a pattern which could also be observed in a combination study employing the HDACi compound VPA together with the virotherapeutic parvovirus vector H-1PV.^22^ In contrast, combination of TSA^16^ and HSV-1 mainly induced proliferation/cell cycle arrest by induction of p21.^35^ Other HDACi/OV combination studies found an enhanced therapeutic effect by induction of oxidative stress,^22^ whereas combination of vorinostat with VSV resulted in an induction of autophagy via modulation of NF-κB signaling.^36^ Finally, addition of TSA to oncolytic treatment with HSV resulted in antiangiogenic effects indicated by a reduction in secretion of VEGF.^21^

Enhanced oncocytotoxic effects of epi-virotherapeutic treatment modalities have frequently been associated with a facilitation of virus replication and spread,^17,22,35^ which seems to be dependent on dosing schedules. Thus, HSV replication could be enhanced by HDACi pretreatment but not by simultaneous cotreatment.^18^ Nevertheless, no influence on primary infection rates as well as on virus replication and spread was found in a setting of TSA plus HSV-1 (G47∆)^21^ in different human proliferating endothelial cells and cancer cell lines. When testing a larger panel of HDACi, some, but not all HDACi, were found to increase replication of a HSV-1–based virotherapeutic in breast cancer cells.^20^

Additional combination approaches applying adenoviral virotherapeutics revealed further mechanistic insights into the complex interactions of OVs with HDACi, as adenovirus receptor CAR was found to be upregulated in presence of HDACi resulting in enhanced primary infection rates. Interestingly, ongoing HDAC inhibition then resulted in antagonistic interactions and was found to diminish adenovirus replication and spread.^24,37^ Recapitulating those findings, influences on OV replication and spread by coadministration of HDACi seem to be multifactorial, and no general rules can be applied, as outcomes seem to be greatly dependent on the specifics of the oncolytic vector system under evaluation and appertaining replication machineries as well as the particular HDACi compound being under investigation.

Here, we report that cotreatment with resminostat, a novel oral HDACi, did not negatively influence MeV-SCD replication and spread but was found to increase rates of primary infection of human hepatoma cells. Hereby, the moderate effects on enhanced virus entry did not translate into increased viral titers in the replication assays. Furthermore, MeV RNA synthesis and assembly require a plethora of host factors, *e.g.*, heat-shock-protein 72, casein kinase II, peroxiredoxin 1, several unidentified kinases, and structural cytoskeletal proteins such as actin.^38^ These elements could all be possible intersections with the pleiotropic activities of HDACi compounds such as resminostat but have not been further elucidated so far. Accordingly, further work has to bring light into these interplays for the respective combination of MeV-SCD with resminostat in hepatoma cells and preferably also in other tumor cell entities.

Additionally, we did not observe any enhanced or decreased oncolytic effect when varying the timing of resminostat application (*e.g.*, adding resminostat before or after infections with MeV-SCD; data not shown). Accordingly, we were able to reason that resminostat treatment hardly interacts with the process of measles vaccine virus replication, at least in cancer cells lacking a sufficient antiviral program.

Variations in MeV-induced cell cytotoxicity among different cancer entities/cell lines seem to be dependent on a multitude of (independent) factors, including virus-specific as well as tumor cell-specific biology, which have lately been characterized by Noll *et al*.^27^ for our study virus MeV-SCD on the NCI-60 tumor cell panel. In this work, primary resistance phenomena were observed for about 40% of the tested tumor cell lines, but not all resistant tumor cell lines were able to induce an antiviral state via the IFN-signaling pathway, clearly indicating the existence of further determinants being involved in the variation of tumor cell line–specific MeV-mediated cytotoxicities. In this context, it is of interest that Lampe *et al*.^28^ identified MeV-SCD–mediated oncolysis not to be solely dependent on functionally intact apoptotic pathways, a finding which underlines the diversity of the complex virus–tumor cell interactions leading to cancer-cell destruction. In another work, Berchtold *et al*.^11^ found high expression levels of the measles entry receptor CD46 on tumor cell surfaces well correlating with high primary infection rates of our MeV virotherapeutics. However, this feature was not found to apply to our combinational therapy regimen (Res + MeV): hepatoma cell–specific expression levels of CD46 were not found to be enhanced by resminostat cotreatment (data not shown). Another point that one has to consider is the diverse genetic equipment distinguishing even our small panel of hepatoma cells (*e.g.*, p53 expression is not altered in HepG2 cells; no expression of p53 can be detected for Hep3B cells, and PLC/PRF/5 cells exhibit reduced p53 levels^39^), leading to distinct rates of apoptosis or altered regulation of cellular protein biosynthesis following virus infection.

Among the multitude of genetic and epigenetic alterations which tumor cells acquire in the process of carcinogenesis, leading to independence of apoptotic signals, unrestricted proliferation, and concealment of tumor cells from immune responses, some are responsible for a disruption of tumor pathways required for sufficient innate immunity signaling, making them preferred targets for OVs. In the context of human hepatoma cell infections, we found that MeV, when used at low MOIs, was not able to induce any detectable amounts of IFN-β, an inflammatory cytokine with a central role in the cellular antiviral repertoire,^40^ indicating defects in pathogen recognition. A screening on the NCI-60 tumor cell panel revealed that about 75% of tumor cell lines were found to have defective IFN responses.^13,41^ However, the same does not necessarily apply to primary tumor cells^13^ and in-patient situations, in which primary resistance phenomena toward oncolytic virotherapy have been observed in various clinical trials. Measles vaccine virus strains (such as the Edmonston strain used in this work), but not wild-type measles virus, induce production of type I IFN in monocyte-derived dendritic cells and peripheral blood lymphocytes.^8,9^ Therefore, it is likely that monocyte-derived dendritic cells being part of the tumor microenvironment are responsible for a sturdy production of type I IFN, inducing (via paracrine secretion processes) an antiviral response in tumor cells lacking virus recognition. On the other hand, inhibition of different components of the IFN response has previously been shown to increase virus replication as well as virus yield in tumor cell cultures.^42^ Therefore, combination strategies of OV and IFN-blocking agents potentially could help to overcome such limitations for the in-patient situation.

To address this heterogeneity in tumor cell susceptibility toward oncolytic virotherapy, HDACi can be employed to repress innate immunity signaling with restriction to malignant cells.^43^ Interestingly, HDACi were found to have the potency to undermine antiviral immunity resulting in enhanced OV replication.^13^ Mechanistically, inhibition of HDAC activity by TSA was found to inhibit IFN-β production, and silencing of HDAC6 was correlated with an increased replication of VSV.^44^ Beyond those findings, HDAC inhibition with TSA/VPA resulted in an impairment of ISG expression after exogenous stimulation with IFN-β without altering activation of STAT proteins and ISGF3 formation.^45^ In detail, HDAC1 was found to associate with STAT1 and STAT2, and inhibition by HDACi leads to a diminished transcription in response to IFN-α.^46^ To summarize those observations, treatment with HDACi has been found to inhibit both IFN secretion and transcriptional activity of several IFN-stimulated genes. In line with these discoveries, we here investigated the potential of resminostat as a known potent inhibitor of class I and IIb HDAC (including HDAC1 and HDAC6) to inhibit induction of ISG. We showed that resminostat suppressed expression of IFIT-1 in HepG2, Hep3B, and PLC/PRF/5 cells after exogenous IFN-β stimulation, possibly preventing the induction of an antiviral state within hepatoma cells, thereby constituting a possible positive modulator for oncolytic virotherapy in tumor cells exhibiting a residual intact antiviral IFN response (see Supplementary Figure S2). Because of the deficient IFN-response found in our hepatoma panel, the enhanced cytotoxic effects of the epi-virotherapeutic combination approach can therefore not be attributed to the IFN-response (immuno-)modulating effects of resminostat. These are proposed additional benefits expected for the *in vivo* application of Res + MeV and have to be tested next in an immunocompetent animal model system.

The IFIT family of antiviral proteins is found to be induced downstream of IFN stimulation following virus infection^7^ with distinct activities against viral functions: *e.g.*, general inhibition of translation initiation is achieved by interaction of IFIT proteins with eIF3.^5^ Furthermore, IFIT-1 (also known as ISG56) was found to directly bind triphosphorylated RNA, which often occurs in the cytosol during life cycles of RNA viruses.^47^ NF-κB signaling is also central for the activation of innate antiviral programs.^48^ After cytosolic activation upon phosphorylation, NF-κB induces inflammatory cytokines such as type I IFN.^1^

Interestingly, zinc finger protein 64 (zfp64) expression was found to be downregulated following resminostat treatment *in vitro* and *in vivo*.^26^ We here detected no negative impact upon zfp64 mRNA levels when performing hepatoma cell infections with MeV-SCD, indicating that resminostat-mediated effects are not impaired in the Res + MeV combination treatment setting. Of note, zfp64 has previously been found to be a positive modulator of NF-κB–mediated signaling following Toll-like receptor–activated inflammatory response in macrophages, and zfp64 knockdown was associated with the inhibition of Toll-like receptor–triggered production of IFN-β, TNF-α, and IL-6.^49^ Therefore, resminostat-mediated immunomodulation can be linked to this NF-κB–dependent innate immunity signaling pathway throughout zfp64 downregulation as well.

The intention of combining an OV with an HDACi is to generate a balanced treatment modality between temporary immunomodulation, favoring OV replication in cancer cells, and a maximum boosted host antitumor adaptive immune response through release of specific tumor antigens following measles virus infection. Our data lead to the conclusion that combination therapies of the novel oral HDACi resminostat with the oncolytic measles vaccine virus MeV-SCD bear a great potential for patients with advanced hepatocellular carcinomas (HCCs), especially as both agents are currently under clinical investigation, which could allow a fast translation of our results “from bench to bedside.” The full benefit of the combination therapy of both compounds for those HCC patients may be even considerably larger, as resminostat *per se* could function as an immunomodulating compound, which potentially could suppress cellular innate antiviral responses in hepatoma cells being refractory to virotherapy, leading to higher concentrations of viral vectors at the respective tumor sites. Based on these promising results, we are currently designing a first epi-virotherapeutic clinical trial employing resminostat together with MeV-SCD in patients exhibiting advanced stages of HCC.

## Materials and Methods

### Cell culture

Human hepatoma cell lines HepG2 (DSMZ-No: ACC-180) and Hep3B (DSMZ-No: ACC-93) were purchased from German Collection of Microorganisms and Cell Cultures (DSMZ, Braunschweig, Germany). PLC/PRF/5 human hepatoma cells were received from the European Collection of Cell Cultures (ECACC, Salisbury, UK; catalogue no: 85061113). VERO-B4 cells (African green monkey; DSMZ-No: ACC-33) were also obtained from DSMZ. Hep3B and PLC/PRF/5 cells were cultured in Dulbecco’s modified Eagle’s medium (DMEM; Sigma Aldrich; Munich, Germany) supplemented with 10% fetal bovine serum (FBS, Biowest, Nuaillé, France). HepG2 cells were cultured using a minimum glucose DMEM (Sigma Aldrich) supplemented with 10% FBS and l-glutamine (10 ml/l). Hepatoma cells were stored in an incubator at 37 °C in a humidified atmosphere enriched with 5% CO_2_. Stimulation with human IFN-β (IFN-β; Pepro-Tech, Rocky Hill, NJ) was achieved by adding 1,000 U/ml IFN-β to the culture medium.

### Propagation and titration of measles vaccine virus

Construction of recombinant measles vectors MeV-GFP (measles vector encoding for GFP as a marker gene integrated into the viral genome) and MeV-SCD (encoding for suicide gene SCD^50^) has been described elsewhere.^11^ Production and propagation of measles vaccine viruses were performed using Vero cells as an optimal virus growth system. For this purpose, 1 × 10^7^ Vero cells were seeded in 15-cm plates, washed once after 24 hours with phosphate-buffered saline (PBS; Sigma Aldrich), and infected for 3 hours at a MOI of 0.03 in infection medium (Opti-MEM; Gibco; Grand Island, NY). Subsequently, medium was changed to DMEM containing 10% FBS. After an incubation period of 54 hours and after microscopy had assured maximum levels of infection, medium was removed, and attached Vero cells were scraped into 1 ml Opti-MEM. Release of virus was achieved by freeze-thaw lysis. After centrifugation (1,900*g*, for 15 minutes at 4 °C), supernatants were stored at −80 °C. Viral titers were determined by using theTCID_50_ (tissue culture infective dose 50) endpoint titration according to Spearman^51^ and Kärber^52^ on Vero cells. Results were converted into plaque-forming units/ml (pfu/ml). Immunofluorescence staining was performed to detect infected cells. For this purpose, cells were washed with PBS, fixed with 50 μl of 4% paraformaldehyde (Otto Fischar, Saarbrücken, Germany) for 10 minutes and subsequently washed two times with PBS. After blocking with 1% FBS in Tris-buffered saline containing 0.02% Tween 20 (TBS-T), the primary antibody MeV N-Protein NP clone 120 Mouse IgG2 (ECACC, diluted 1:1,000) was added for 30 minutes at room temperature. After washing three times with TBS-T, the incubation with the secondary antibody (Alexa Fluor 546 Goat AntiMouse IgG (H+L), A11003; Invitrogen, Carlsbad, CA; diluted 1:1,000) was performed for 30 minutes in the dark. After three final washing steps (TBS-T), the plates were analyzed via fluorescence microscopy using a standard IX50 fluorescence microscope (Olympus, Tokyo, Japan).

### Infection of cells with measles vaccine virus

Cells were plated the day before infection. After washing with PBS, cells were infected with varying MOIs of MeV-GFP or MeV-SCD diluted in Opti-MEM. Three hours postinfection, the inoculum was removed and replaced with DMEM or DMEM containing resminostat (provided by 4SC AG, Planegg-Martinsried, Germany) at indicated concentrations.

### SRB cytotoxicity assay

To quantify cytotoxicity of compounds under investigation, we used the Sulforhodamine B assay.^53^ Human hepatoma cells (2–4 × 10^4^/well) were cultured in 24-well plates and then treated as described above. At 96 hpi, hepatoma cells were washed with cold PBS and fixed with 10% trichloracetic acid for 30 minutes at 4 °C. After washing the plate with tap water and subsequent drying, tumor cells were stained with SRB dye (Sigma, 0.4 in 1% acetic acid); unbound dye was removed by washing with 1% acetic acid. Protein-bound dye was brought into solution with 10 mmol/l Tris base (pH 10.5), and optical densities were determined using a microtiter plate reader (Tecan Genios Plus; Tecan Deutschland, Crailsheim, Germany) at a wavelength of 550 nm.

### CellTiter-Blue cell viability assay

The CellTiter-Blue cell viability assay was used as a more functional endpoint measurement to confirm SRB data (exemplarily for HepG2 cells). HepG2 hepatoma cells (4 × 10^4^/well) were cultured in 24-well plates and then treated as described above. To ensure equal amounts of culture medium per well, the supernatants of all wells of the same condition were pooled in a 2-ml reaction tube, and 200 μl were readded per well. The admixture of 40 µl CellTiter-Blue reagent (Promega, Madison, WI) per well started the incubation time that had to be empirically determined and took 1 hour for HepG2 cells. Read-out was performed on the microtiter plate reader Tecan Genios Plus (Tecan) with an excitation filter of 584 nm and run under the XFluor software.

### Real-time cell proliferation assay

HepG2 (10^4^ cells/well) were seeded in 96-well plates (E-Plate 96, ACEA Biosciences, San Diego, CA). Real-time dynamic cell proliferation was monitored in 30-minute intervals for over 120 hours using the xCELLigence SP system (Roche Applied Science, Mannheim, Germany). After 21 hours, cells were treated with MeV and after additional 3 houts with resminostat, as indicated. Cell index values were calculated using the RTCA Software (1.2.1.1002). All curves were normalized to the time point after the resminostat treatment was conducted (~24 hours after seeding) applying the RTCA Software.

### Quantification of primary infection rates

Human hepatoma cells (1.5–4 × 10^5^/well) were cultured in six-well plates and then infected with a measles vaccine virus encoding a GFP marker gene (MeV-GFP) at MOI 1 and treated with resminostat at 1 μmol/l. At 24 hpi, hepatoma cells were washed once with 2 ml PBS/well and detached with 0.5 ml Accutase (PAA Laboratories, Cölbe, Germany). Subsequently, Accutase was inactivated with 2 ml FACS buffer (PBS plus 10% FBS). Tumor cells were washed with PBS. After centrifugation (302*g*, 5 minutes), the tumor cell pellet was resuspended in FACS buffer plus 4% paraformaldehyde (Otto Fischar). Differences in rates of primary infection were analyzed on the FACSCalibur cytometer (Becton Dickinson, Franklin Lakes, NJ) and digitally processed with the CellQuest software (Becton Dickinson).

### Viral growth curves

To compare the kinetics of viral growth in our hepatoma cell lines after infection with MeV-SCD alone or in combination with resminostat, viral growth curve assays were performed. For this purpose, human hepatoma cells were plated 24 hours before infection in 24-well plates. Cells were infected with different MOIs, as indicated. Three hpi, after washing the plates three times with PBS, the inoculum was substituted with DMEM or DMEM containing resminostat. At 3, 24, 48, 72, and 96 hpi, supernatants and cells (being scraped into 0.5 ml Opti-MEM) were harvested. Following a single freeze-thaw cycle, quantification of virus titers was performed on Vero cells as described above.

### Analysis of cell cycle profiles by flow cytometry

For this assay, human hepatoma cells (2–4 × 10^4^/well) were again cultured in 24-well plates and infected with MeV-SCD the next day at indicated MOIs in Opti-MEM. Three hours postinfection, medium was changed to DMEM (MOCK) or DMEM containing 1 μmol/l resminostat. After an incubation period of 96 hours, cells were stained using the Nicoletti staining protocol^54^: both cell culture medium as well as PBS, which was used to wash the plates, were collected together with the cells, being detached by using trypsin. Cells were centrifuged at 300*g*. The cell pellet was resuspended in a hypotonic propidium iodide buffer (1 mg/ml sodium citrate; 0.003 ml/l Triton X-100; 0.02 mg/ml ribonuclease A; 0.1 mg/ml propidium iodide, filled up with double distilled water), and cells were incubated for 30 minutes in the dark. As the buffer solubilizes plasma membranes and RNAse digests intracellular RNA, propidium iodide as an intercalating nucleic acid–binding fluorescent dye interacts exclusively with intracellular DNA in a proportional manner. As the intracellular DNA content is dependent on the stages of the cell cycle, cellular DNA content indicates in which phase of the cell cycle the trespassing cell currently is (*e.g.*, hypoploid signals are detected in apoptotic cells). Fluorescence signals were detected on a FACSCalibur flow cytometer (Becton Dickinson), and data analysis was performed using FLOWJO flow cytometric analysis program (FLOWJO, Ashland, OR).

### Analysis of the mitochondrial transmembrane potential Δψm by TMRE staining

To gain further mechanistic insight into the proposed enhanced apoptotic rate promoted by our combinational treatment, we selected HepG2 cells to exemplarily analyze breakdown on mitochondrial transmembrane potential as an indicator for ongoing apoptotic processes using the TMRE staining assay. Accordingly, HepG2 human hepatoma cells (5 × 10^5^/well) were cultured in six-well plates and infected with MeV-SCD the next day at MOI 0.1 in Opti-MEM. Three hpi, medium was changed to DMEM (MOCK) or DMEM containing 1 μmol/l resminostat. After an incubation period of 96 hours, the mitochondrial transmembrane potential Δψm was determined by TMRE staining. Both cell culture medium as well as PBS, which was used to wash the plates, were collected together with the cells, being detached by using trypsin. Cells were centrifuged at 200*g* and washed once with PBS. For staining, cells were incubated in PBS containing 100 nmol/l TMRE (Molecular Probes, Leiden, The Netherlands) for 20 minutes at 37 °C. Then, 5 ml PBS were added, and cells were centrifuged again. The cell pellet was resuspended in PBS supplemented with 1% fetal calf serum. Flow cytometric analysis was performed on a FACSCalibur flow cytometer (Becton Dickinson) using Cell Quest Software (Becton Dickinson). Unstained control cells were used as a reference for TMRE-negative cells.

### Immunoblotting

Human hepatoma cells were seeded in six-well plates (1.5–4 × 10^5^/well), and the next day stimulated with 1,000 U/ml IFN-β and/or treated with 5 μmol/l resminostat. Another 24 hours later, cells were washed with PBS and transferred into lysis buffer (50 mmol/l Tris, 150 mmol/l NaCl, 1% IGEPAL CA-630 (Sigma-Aldrich)). A total of three freeze-thaw cycles was followed by centrifugation to remove cell debris. To quantify the amounts of total protein in the supernatants, Bradford Protein assays (Bio-Rad Laboratories, Hercules, CA) were carried out. A total of 50-μg protein was separated by 8% sodium dodecyl sulfate–polyacrylamide gel electrophoresis and transferred to a hydrophobic polyvinylidene difluoride membrane (Amersham Hybond-P; GE Healthcare, Buckinghamshire, UK). Use of a prestained PageRuler Plus protein ladder (Thermo Scientific, Waltham, MA) allowed the determination of molecular weights. Blocking in 5% powdered milk (Carl Roth, Karlsruhe, Germany) in TBS-T was followed by incubation with the primary antibodies (anti-IFIT1: GTX103452; 1:1,000; GeneTex, Irvine, CA; anti-Phospho-STAT1: 58D61; 1:1,000; Cell Signaling Technology, Danvers, MA; anti-STAT1: sc-591; 1:500; Santa Cruz Biotechnology, Santa Cruz, CA; anti-β-actin: A 4700; 1:5,000; Sigma Aldrich). After washing the membranes three times with TBS-T, secondary horseradish peroxidase–coupled antibodies (goat antirabbit IgG; goat antimouse IgG; HRP-coupled; Abcam, Cambridgeshire, UK) were added for 1 hour. Visualization of protein bands was performed using enhanced chemiluminescence western blotting detection reagent kit (GE Healthcare) and a high performance chemiluminescence Amersham Hyperfilm enhanced chemiluminescence (GE Healthcare).

### qPCR

RNA was isolated on the NucleoSpin RNA kit (Macherey-Nagel, Düren, Germany) according to the manufacturer’s instructions. 500 ng of each RNA sample were mixed with 2 µl M-MLV RT buffer (Promega, Madison, WI), 1 µl RNase-inhibitor RNasin Plus (Promega), 1 µl oligo-dT-Primer (TIB MolBio, Berlin, Germany), 0.5 µl dNTP mix (Roti-Mix PCR3, Carl Roth) and added up to a total volume of 9.6 µl in RNAse-free water. Samples were then incubated at 70 °C for 2 minutes. After adding 0.4 µl reverse-transcriptase M-MLV RT H(-) Point Mutant (Promega), samples were incubated at 42 °C for 60 minutes.

The cDNA samples were diluted (1/20) with tRNA-H_2_O; primers were used in a concentration of 500 nmol/l. PCR was carried out in an iCycler (BioRad) with iQ5 Multicolor Real-time Detection System (BioRad), using the following setup: 10 µl iQSYBR Green PCR Master Mix (Promega), 0.1 µl of each primer (100 µmol/l stock), 7.8 µl H_2_0, and 2 µl cDNA (diluted 1/20). The following primer pairs were used: zfp64 (splicing variants 1,3,4) forward: ACCTGCCCACGGAAAGTAAT; zfp64 (splicing variants 1,3,4) reverse: TATGGGGTTTGTCTCCCGTG; RPS18 (housekeeping gene) forward: GAGGATGAGGTGGAACGTGT; RPS18 reverse: TCTTCAGTCGCTCCAGGTCT. PCR was carried out with the following thermal profile: 3 minutes at 95 °C with subsequently 40 cycles for 15 seconds at 95 °C, 20 seconds at 60 °C, and 15 seconds at 62 °C. Heating up for 1 minute at 95 °C was followed by 1 minute at 65 °C and 81 cycles at 65 °C cooling down to 20 °C. Target gene expression was evaluated via the 2^-ΔCt^ method and normalized to the housekeeping gene *RPS18* and subsequently graphed relative to the respective MOCK sample for each time point and expressed as “relative gene expression.”
