# Supplementary material for: Combination of the oral histone deacetylase inhibitor resminostat with oncolytic measles vaccine virus as a new option for epi-virotherapeutic treatment of hepatocellular carcinoma
Source: Mol Ther Oncolytics. 2015 Oct 7;2:15019–. doi: 10.1038/mto.2015.19 (PMC4782956; doi:10.1038/mto.2015.19)
Supplement: Supplementary Figures [file mto201519-s1.doc]

# Supplementary Figures

**Supplementary Figure 1 Additional set of experiments** **carried out exemplarily for HepG2 tumor cells: (A)** CellTiter-Blue Viability assay: Human hepatoma HepG2 cells were infected with MeV-SCD (MOI of 0.1) and co-treated with resminostat (1 μM) at 3 hpi. Endpoint measurements were performed at 96 hpi. Displayed are means and standard deviations of five independent experiments, each carried out in quadruplicates; p-values of one-way ANOVA with a Tukey post-test. **(B)** Analysis of the mitochondrial transmembrane potential Δψm by TMRE staining: Infection and treatment with resminostat of HepG2 cells were performed as described above. Mitochondrial trans­membrane potential Δψm was determined by TMRE staining at 96 hpi. Displayed are means and standard deviation of three independent experiments, p-values of one-way ANOVA with a Tukey post-test. **(C)** Real-time cell monitoring of HepG2 hepatoma tumor cells over a period of 120 hours: HepG2 cells were infected with MeV-SCD (single agent, red curve) or mock infected (black curve) and subsequently treated with resminostat alone (blue curve) or in a combination setting (MeV + Res, purple curve) as in (A) before measurement. Triton X-100 0.1 % was used as a negative control (white curve). Cellular impedance was measured continuously using the xCELLigence SP system. Cell index values and SD of four independent experiments obtained every 5 hours are displayed. **(D)** Cellular impedance displayed as normalized cell index for HepG2 cells, 120 hpi (frame in (C)). Res: resminostat. MeV: suicide gene-armed measles vaccine-based virotherapeutic MeV-SCD. MOI: multiplicity of infection. hpi: hours post infection. n.s.: not significant (p>0.05).

**
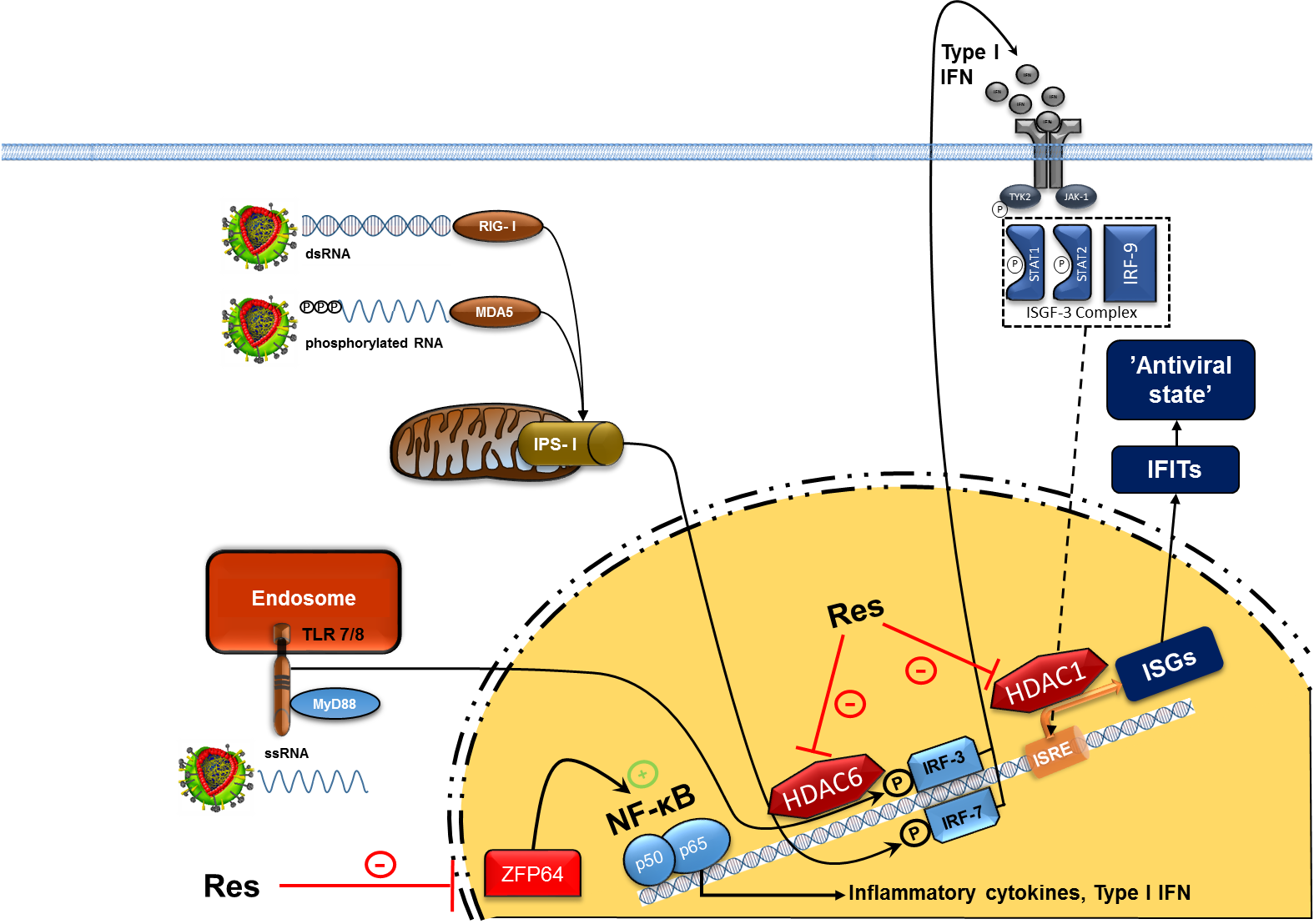
**

**Supplementary Figure 2: Possible influences of resminostat on interferon (IFN) signaling**: Virus derived pathogen-associated molecular patterns (PAMPs), such as ssRNA, dsRNA or triphosphorylated RNA are recognized e.g. by cytosolic receptors, such as RIG-I/MDA5 or TLRs followed by signaling cascades resulting in NF-κB or IRF-3/IRF-7 activation and translocation to type I IFN promoter sites. Subsequently, newly synthesized inflammatory cytokines (such as type I IFN) bind to membranous IFN-receptors, leading to phosphorylation of STAT1 & STAT2, which form a heterotrimeric ISGF-3 complex together with IRF-9. ISGF-3 complex binding to the IFN-stimulated response element (ISRE) promoter sites triggers ISG (interferon-stimulated genes) production, such as IFIT (interferon-induced protein with tetratrico­peptide repeats)-family proteins, which are responsible for induction of an antiviral state within the infected cell. HDACs are involved in IFN production and induction of ISGs. Resminostat is an inhibitor of HDAC1 & HDAC6 and is responsible for downregulation of ZFP64, a positive regulator of NF-κB signaling. Res: resminostat; dsRNA: double stranded RNA; ssRNA: single-stranded RNA; RIG-I: retinoic acid-inducible gene 1; MDA5: melanoma-differentiation-associated protein 5; IPS-1: IFN-β promoter stimulator 1; TLR: Toll-like receptor; MyD88: myeloid differentiation primary response gene 88; HDAC: histone deacetylase; IRF: IFN regulatory factors; IFN: interferon; STAT: signal transducers and activators of transcription; ISGF: IFN-stimulated gene factor; ISRE: IFN-stimulated response element; ISG: IFN-stimulated gene; IFIT: IFN-induced proteins with tetratricopeptide repeats; NF-κB: nuclear factor kappa-light-chain-enhancer of activated B cells.
